# Supplementary material for: Multiphoton Microscopy Reveals DAPK1-Dependent Extracellular Matrix Remodeling in a Chorioallantoic Membrane (CAM) Model
Source: Cancers (Basel). 2022 May 10;14(10):2364. doi: 10.3390/cancers14102364 (PMC9139596; doi:10.3390/cancers14102364)
Supplement: Supplementary file 1 [file cancers-14-02364-s001.zip › cancers-1699830-supplementary/SI - ECM remodelling potential_V6.4(Cancers revision)_PK.pdf]

cancers

MDPI

Supplementary Information for

Multiphoton Microscopy Reveals DAPK1-Dependent Extracellular Matrix Remodeling in a Chorioallantoic Membrane (CAM) Model

Philipp Kunze†, Lucas Kreiss†, Vendula Novosadová, Adriana V. Roehe, Sara Steinmann, Jan Prochazka, Carol I. Geppert, Arndt Hartmann, Sebastian Schürmann, Oliver Friedrich‡, Regine Schneider-Stock‡

† equally contributing first authors

‡ equally contributing, corresponding authors: Regine Schneider-Stock and Oliver Friedrich
regine.schneider-stock@uk-erlangen.de, oliver.friedrich@fau.de

This PDF file includes:

Figures S1 to S5
Tables S1
Legends for Movies S1 to S2

Other supplementary materials for this manuscript include the following:

Movies S1 to S2

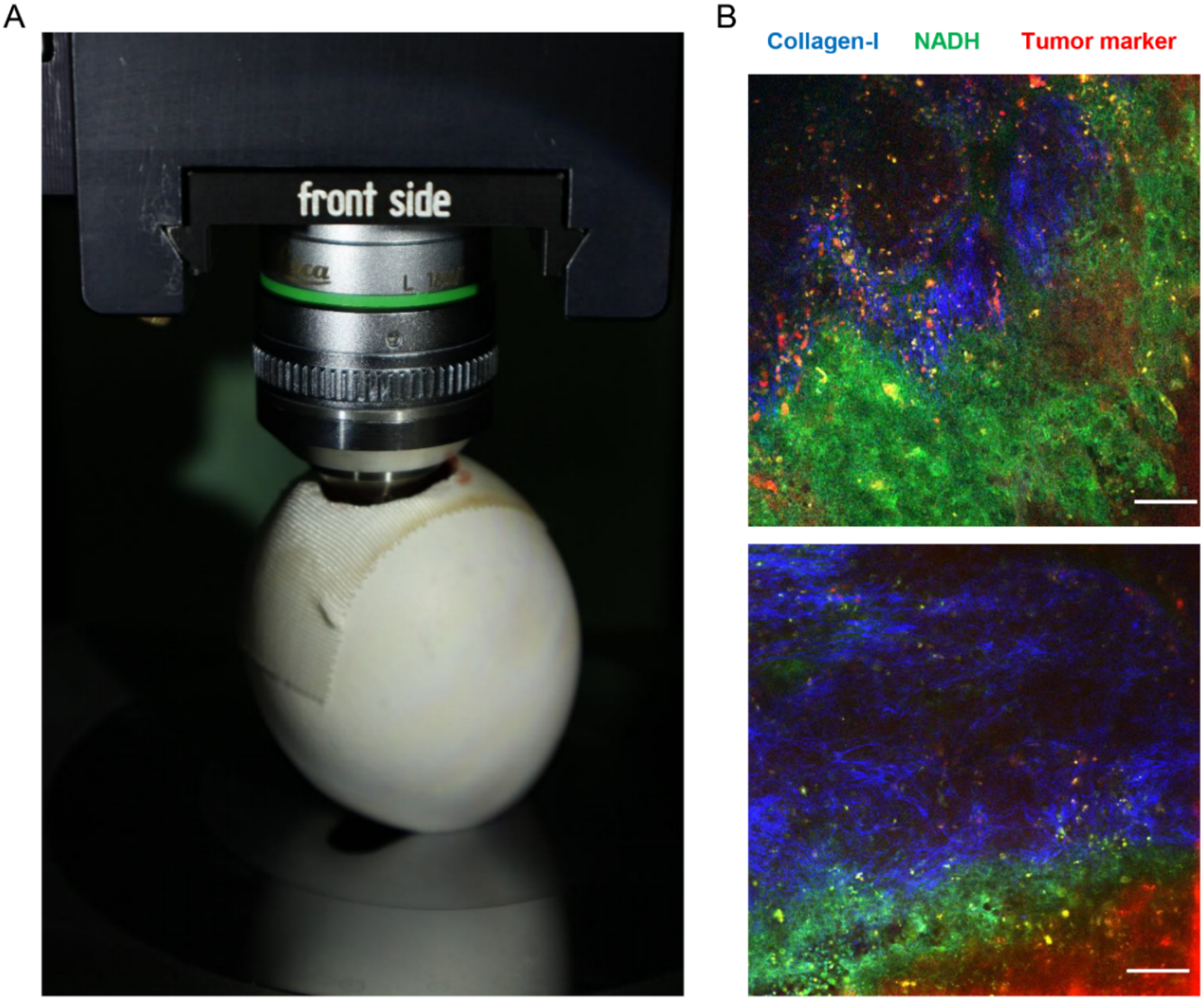

Figure S1. Direct *in ovo* imaging under *in vivo* conditions of the chicken embryo. A) The egg was opened and multiphoton imaging was directly performed via the opening in the egg shell using a 16X objective with large working distance (WD = 8 mm). B) Two representative examples of the invasion front of DAPK1 ko tumor on the CAM as measured directly by *in ovo* multiphoton microscopy (scale bar = 50 µm).

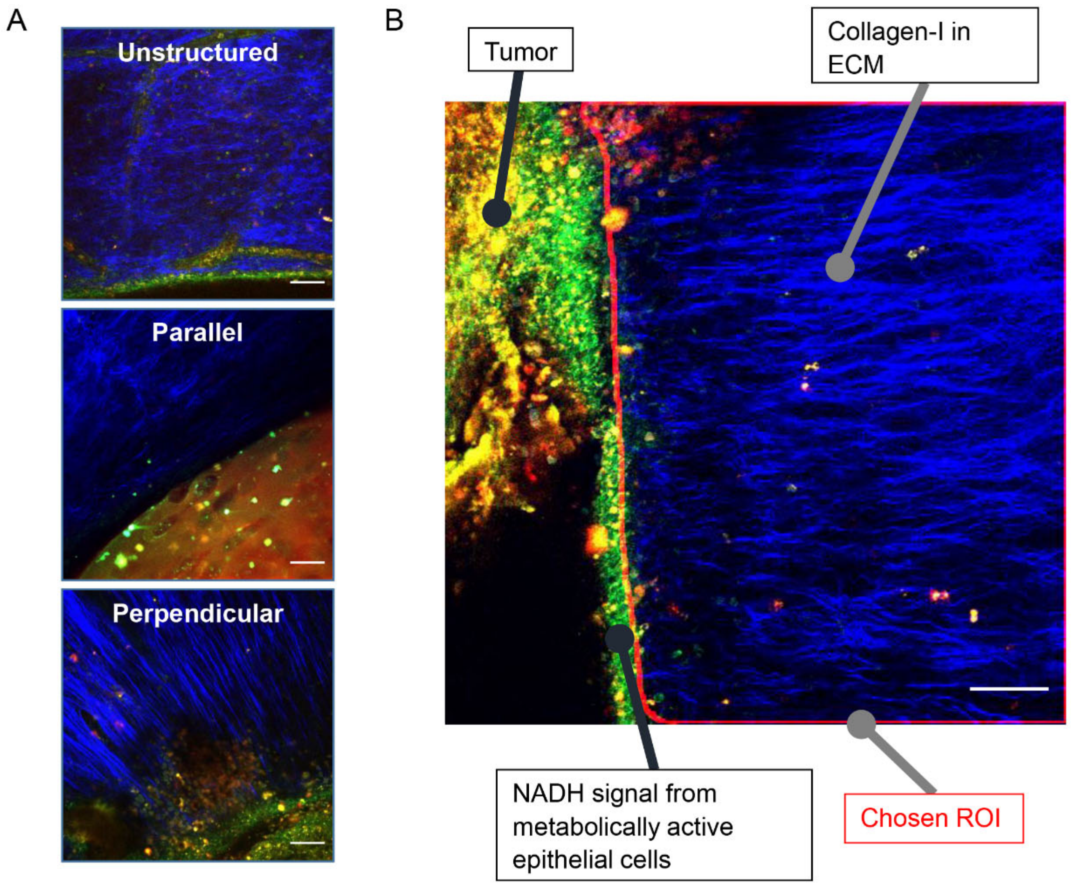

Figure S2. Evaluation criteria for fibrillar collagen orientation and density analysis. A) Example images representing the three classes (unstructured, parallel, and perpendicular) used for the fibrillar collagen orientation analysis (scale bar = 50 µm). B) Criteria for manual selection of the ECM as region of interest (ROI) used for fibrillar collagen density analysis (scale bar = 50 µm).

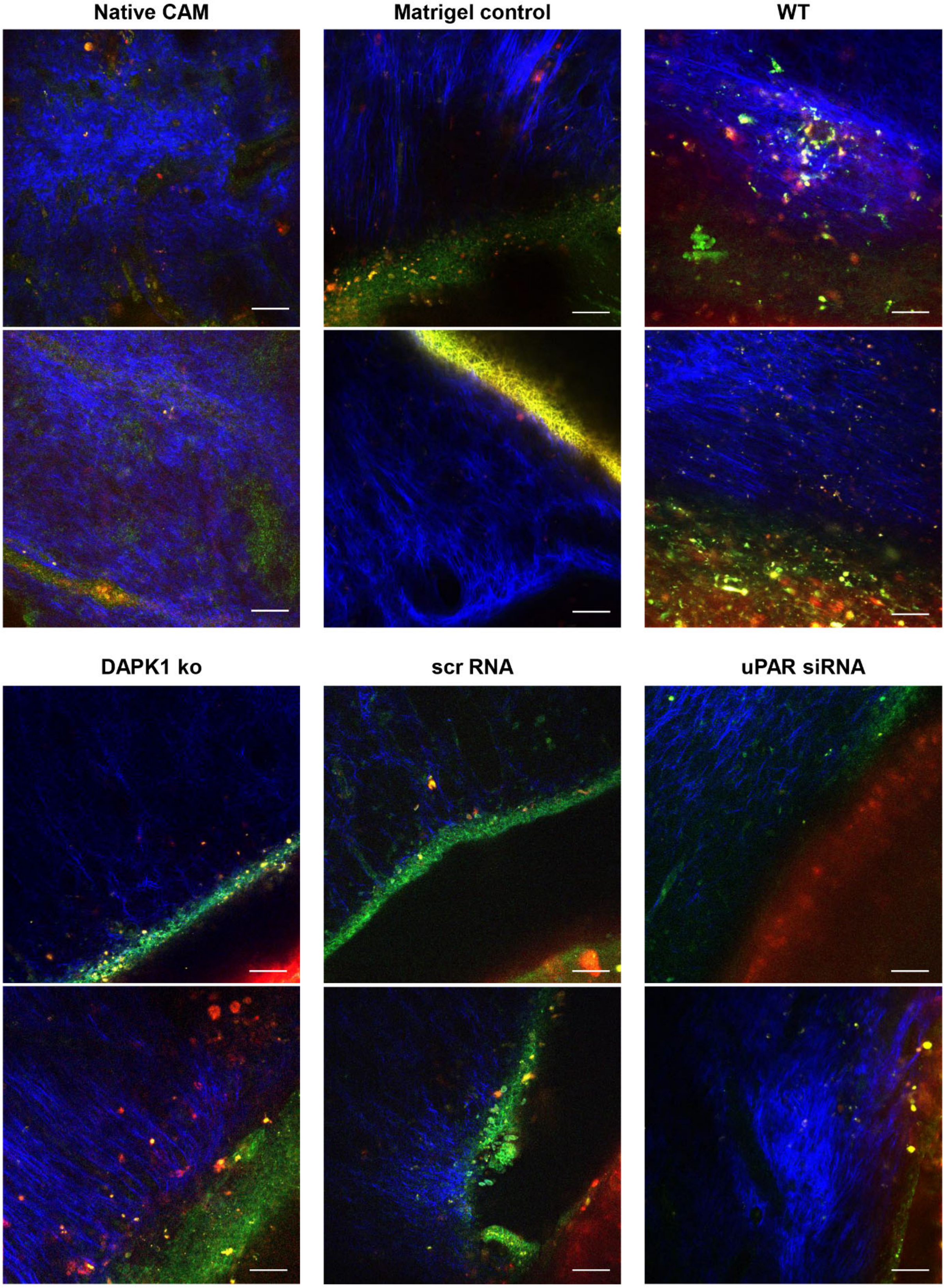

Figure S3. Additional representative examples of the collagen network at the tumor interface of *ex ovo* CAM xenografts under the different tested conditions: Native CAM with an unstructured, Matrigel, DAPK1 ko clone, and scrRNA treated tumor cells with perpendicular, and HCT116 WT and uPAR siRNA-treated tumor cells with parallel fiber orientation (scale bar = 50 μm).

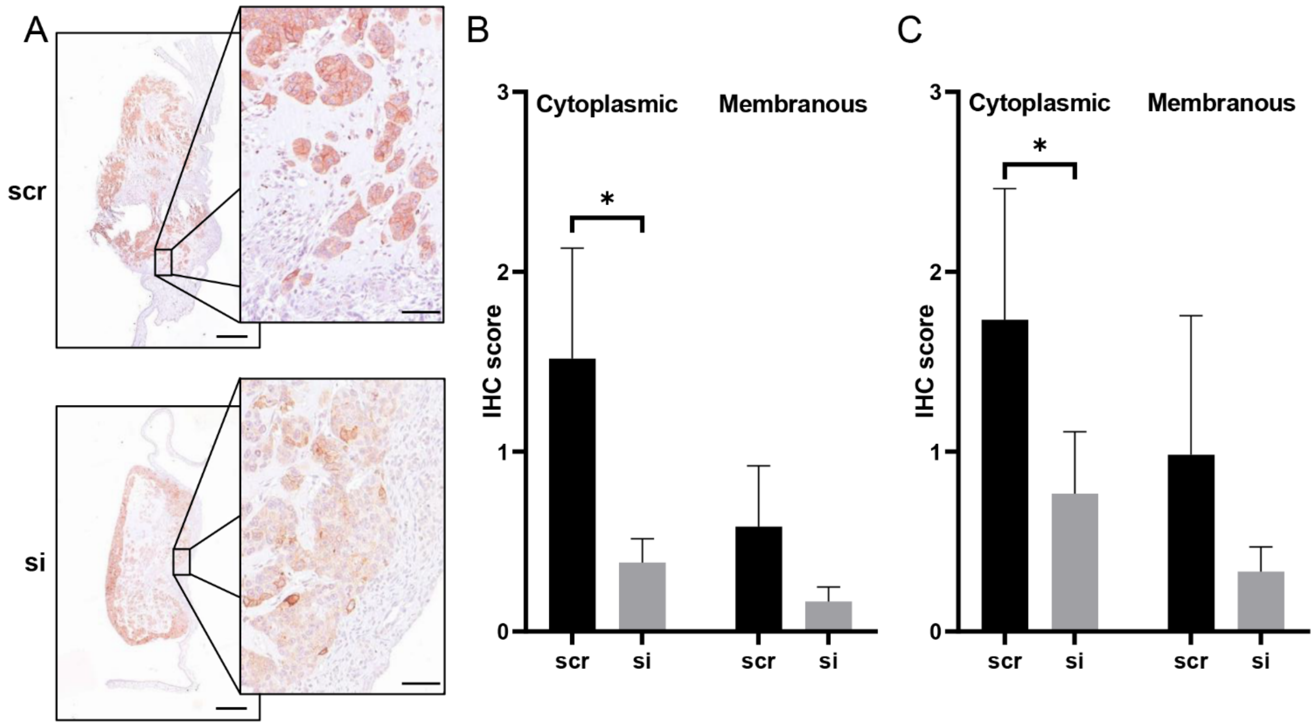

Figure S4. Evaluation of uPAR expression in histological slices of CAM xenografts after uPAR silencing. A) Conventional immunohistochemical stainings for uPAR of CAM xenografts with scrRNA and uPAR siRNA treated HCT116 DAPK1 ko cells. (Scale bar main image = 400 µm; Scale bar zoomed inlet 50 µm).B) Cytoplasmic and membranous uPAR IHC score of HCT116 DAPK1 ko CAM tumors after uPAR silencing at the invasion front and C) at the tumor center ($n_{samples}$ = 6; 2-way ANOVA: *$p < 0.05$). Data represented as mean ± SD.

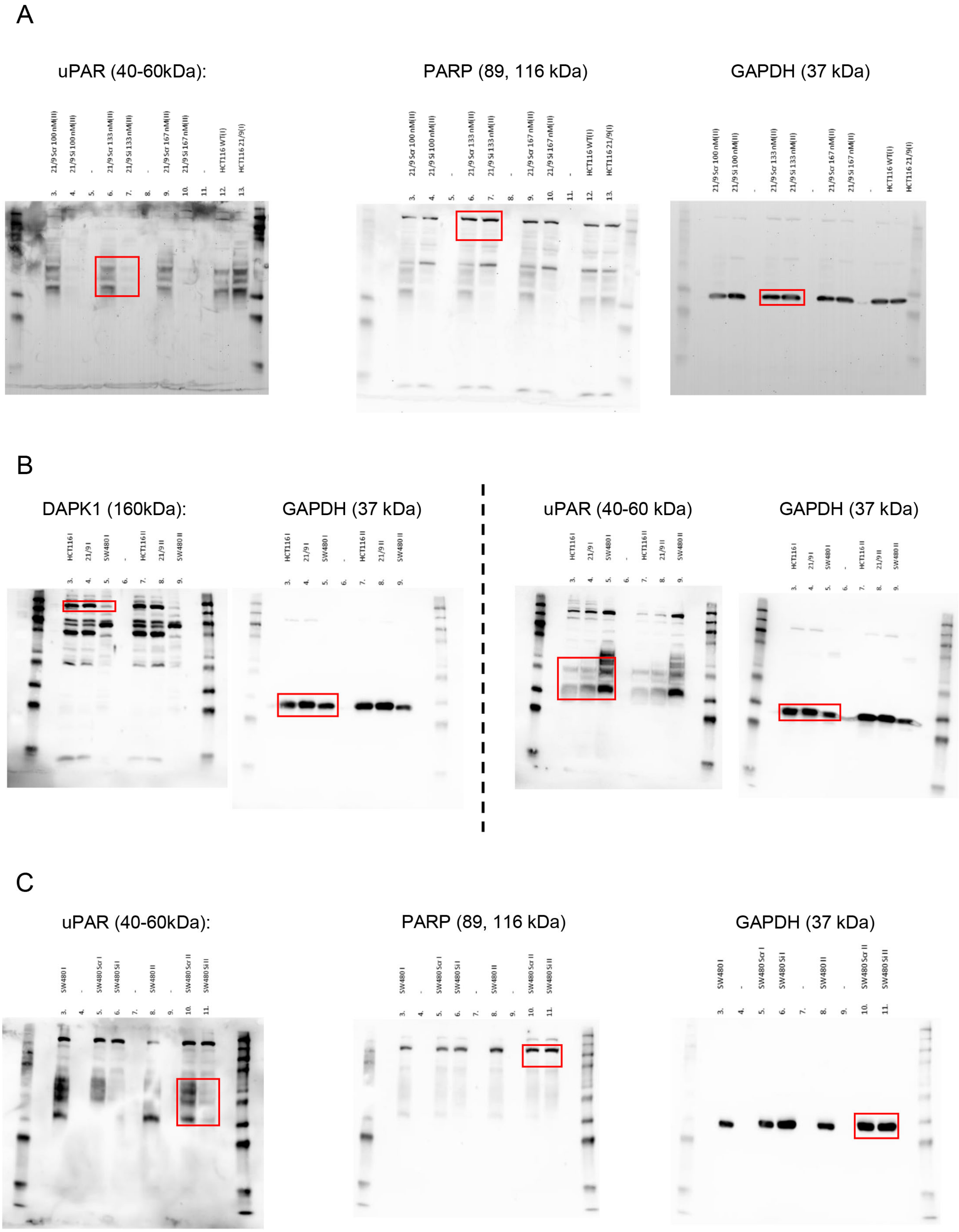

Figure S5. Western blot original data. A) Original Western blot images of blots presented in Figure 4E; B) Figure 5A; C) Figure 5E. Red box represents the shown image selection in the main text.

Table S1. Expression list of GO term annotated proteins that are significantly dysregulated in HCT116 DAPK1 ko cells, which are presented in the GO term network.

Uniprot ID	Gene	Name	log2(FC)	p-value
O14879	IFIT3	Interferon-induced protein with tetratricopeptide repeats 3	4.791	1.589E-10
O15427	SLC16A3	Monocarboxylate transporter 4	1.383	9.723E-03
O60504	SORBS3	Vinexin	-5.119	8.355E-03
O75190	DNAJB6	DnaJ homolog subfamily B member 6	3.306	2.068E-02
O95999	BCL10	B-cell lymphoma/leukemia 10	5.431	1.492E-02
P01130	LDLR	Low-density lipoprotein receptor	-1.042	3.346E-03
P04049	RAF1	RAF proto-oncogene serine/threonine-protein kinase	-2.714	2.564E-02
P04062	GBA	Lysosomal acid glucosylceramidase	-1.247	2.004E-03
P05161	ISG15	Ubiquitin-like protein ISG15	3.522	3.871E-08
P06400	RB1	Retinoblastoma-associated protein	-1.093	2.160E-02
P07737	PFN1	Profilin-1	1.128	2.517E-02
P08582	MELTF	Melanotransferrin	-2.646	3.134E-02
P09601	HMOX1	Heme oxygenase 1	-1.065	5.849E-03
P09914	IFIT1	Interferon-induced protein with tetratricopeptide repeats 1	6.984	2.587E-11
P11166	SLC2A1	Solute carrier family 2	-1.044	3.899E-05
P12109	COL6A1	Collagen alpha-1(VI) chain	-4.297	1.304E-09
P13073	COX4I1	Cytochrome c oxidase subunit 4 isoform 1	1.963	1.926E-02
P13284	IFI30	Gamma-interferon-inducible lysosomal thiol reductase	2.075	1.170E-05
P13747	HLA-E	HLA class I histocompatibility antigen alpha chain E (sHLA-E)]	3.495	2.542E-03
P16220	CREB1	Cyclic AMP-responsive element-binding protein 1	3.422	4.135E-02
P18084	ITGB5	Integrin beta-5	-1.044	7.556E-04
P19525	EIF2AK2	Interferon-induced	1.227	1.147E-04
P23497	SP100	Nuclear autoantigen Sp-100	1.665	4.394E-06
P26583	HMGB2	High mobility group protein B2	1.694	1.844E-02
P29323	EPHB2	Ephrin type-B receptor 2	2.476	2.912E-02
P31327	CPS1	Carbamoyl-phosphate synthase [ammonia]	-1.375	7.847E-04
P35914	HMGCL	Hydroxymethylglutaryl-CoA lyase	-1.188	6.380E-03
P37837	TALDO1	Transaldolase	1.715	1.395E-02
P42224	STAT1	Signal transducer and activator of transcription 1-alpha/beta	2.239	3.300E-08
P43121	MCAM	Cell surface glycoprotein MUC18	-5.997	4.239E-09
P50148	GNAQ	Guanine nucleotide-binding protein G(q) subunit alpha	-1.111	1.431E-02
P52434	POLR2H	DNA-directed RNA polymerases I and III subunit RPABC3	4.372	1.493E-02

P53355	DAPK1	Death-associated protein kinase 1	0.000	0.000E+00
P62328	TMSB4X	Thymosin beta-4	2.611	1.974E-02
P80217	IFI35	Interferon-induced 35 kDa protein	2.933	8.649E-07
Q00978	IRF9	Interferon regulatory factor 9	6.256	8.315E-05
Q01581	HMGCS1	Hydroxymethylglutaryl-CoA synthase	-1.199	1.237E-04
Q01628	IFITM3	Interferon-induced transmembrane protein 3	3.971	1.539E-03
Q01650	SLC7A5	Large neutral amino acids transporter small subunit 1	4.590	1.306E-03
Q02750	MAP2K1	Dual specificity mitogen-activated protein kinase kinase 1	1.293	1.951E-03
Q02880	TOP2B	DNA topoisomerase 2-beta	-3.443	4.768E-02
Q03405	PLAUR	Urokinase plasminogen activator surface receptor	4.020	2.468E-02
Q07973	CYP24A1	1,25-dihydroxyvitamin D(3) 24-hydroxylase, mitochondrial	-4.205	4.421E-03
Q08722	CD47	Leukocyte surface antigen CD47	3.296	3.779E-02
Q10589	BST2	Bone marrow stromal antigen 2	5.461	7.326E-11
Q13153	PAK1	Serine/threonine-protein kinase PAK 1	4.431	2.178E-02
Q13287	NMI	N-myc-interactor	3.446	2.017E-02
Q13308	PTK7	Inactive tyrosine-protein kinase 7	-1.074	1.327E-06
Q14116	IL18	Interleukin-18	1.470	2.472E-04
Q14118	DAG1	Dystroglycan (Dystrophin-associated glycoprotein 1)	-1.408	8.679E-05
Q15154	PCM1	Pericentriolar material 1 protein	-4.521	6.538E-03
Q15532	SS18	Protein SSXT	4.610	2.132E-02
Q15646	OASL	2'-5'-oligoadenylate synthase-like protein	7.160	4.957E-12
Q5ZPR3	CD276	CD276 antigen	-1.043	6.823E-05
Q6P1J9	CDC73	Parafibromin	-1.209	1.004E-02
Q6ZSZ5	ARHGEF18	Rho guanine nucleotide exchange factor 18	2.591	2.606E-02
Q71DI3	HIST2H3A	Histone H3.2	2.079	2.017E-02
Q8IVL6	P3H3	Prolyl 3-hydroxylase 3	3.735	1.963E-03
Q8IXQ6	PARP9	Protein mono-ADP-ribosyltransferase PARP9	6.298	3.489E-04
Q8TDB6	DTX3L	E3 ubiquitin-protein ligase DTX3L	5.544	2.944E-03
Q8WYL5	SSH1	Protein phosphatase Slingshot homolog 1	4.625	1.410E-03
Q96QZ7	MAGI1	Membrane-associated guanylate kinase	1.493	1.779E-05
Q9H4A6	GOLPH3	Golgi phosphoprotein 3	-1.085	5.117E-05
Q9H6T0	ESRP2	Epithelial splicing regulatory protein 2	4.718	1.493E-07
Q9H6U6	BCAS3	Breast carcinoma-amplified sequence 3	2.953	8.656E-03
Q9NQS3	NECTIN3	Nectin-3	-1.156	1.345E-03
Q9UBV2	SEL1L	Protein sel-1 homolog 1	-1.569	2.013E-03
Q9UL46	PSME2	Proteasome activator complex subunit 2	1.247	8.545E-04
Q9Y3D6	FIS1	Mitochondrial fission 1 protein	4.808	1.753E-02
Q9Y6K5	OAS3	2'-5'-oligoadenylate synthase 3 ((2-5')oligo(A) synthase 3)	2.556	2.082E-07

Video S1 (separate file). Exemplary axial scan from an *in ovo* measurement of the CAM with living chicken embryo. Motion artefacts can be observed during life *in ovo*/*in vivo* measurement. The scan starts at the upper surface of the CAM tissue (z_0 = 0 µm) and ends in a depth of z = 216 µm. The spacing between adjacent optical sections is 2 µm. Scale bar = 50 µm.

Video S2 (separate file). Exemplary 3D renderings for native CAM, Matrigel control, HCT116 WT and HCT116 DAPK1 ko tumors.
