# Supplementary figures and images for: Multiphoton Microscopy Reveals DAPK1-Dependent Extracellular Matrix Remodeling in a Chorioallantoic Membrane (CAM) Model
Source: Cancers (Basel). 2022 May 10;14(10):2364. doi: 10.3390/cancers14102364 (PMC9139596; doi:10.3390/cancers14102364)

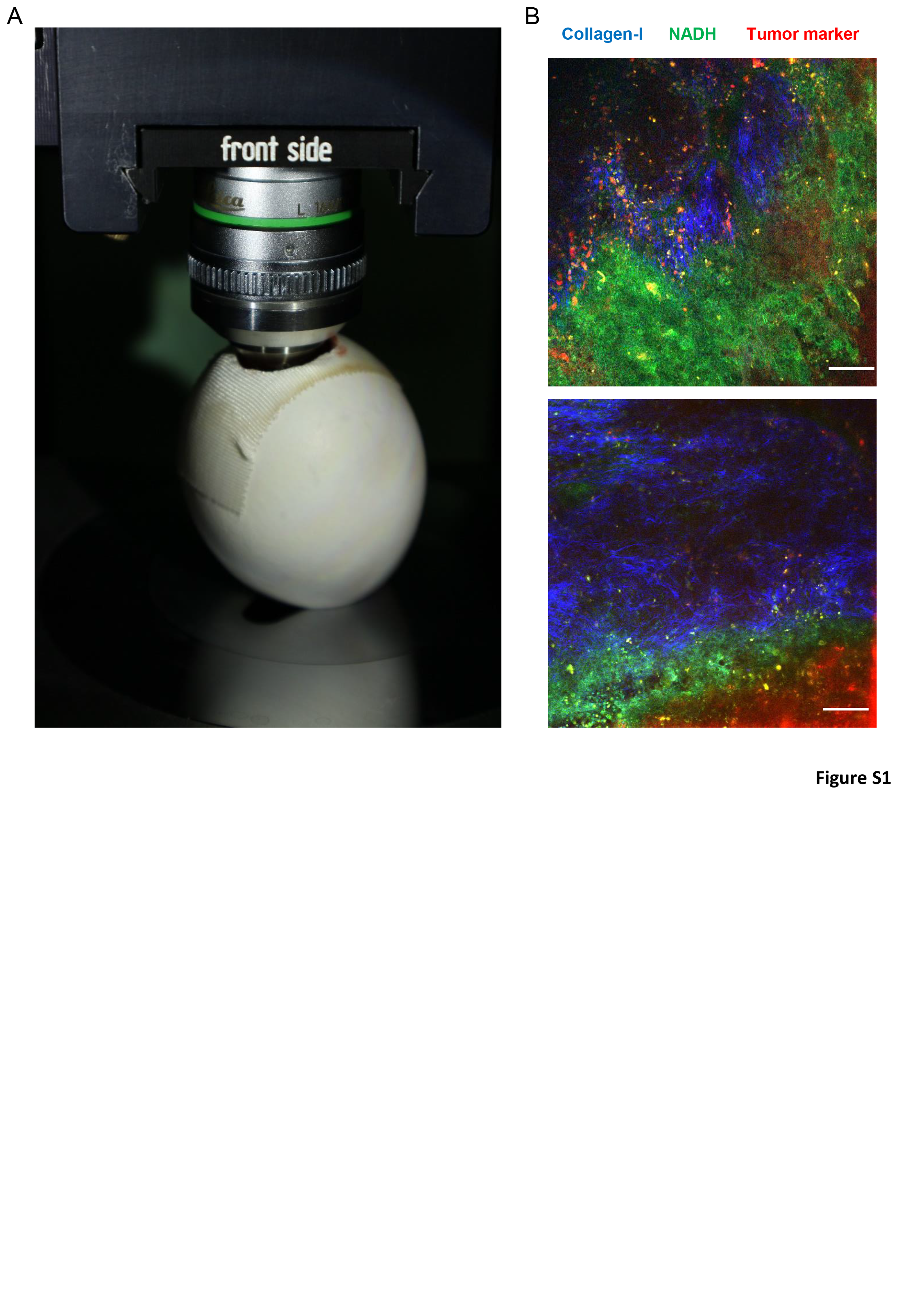

Supplement: Supplementary file 1 [file cancers-14-02364-s001.zip › cancers-1699830-supplementary/Figure S1(300dpi).tif]

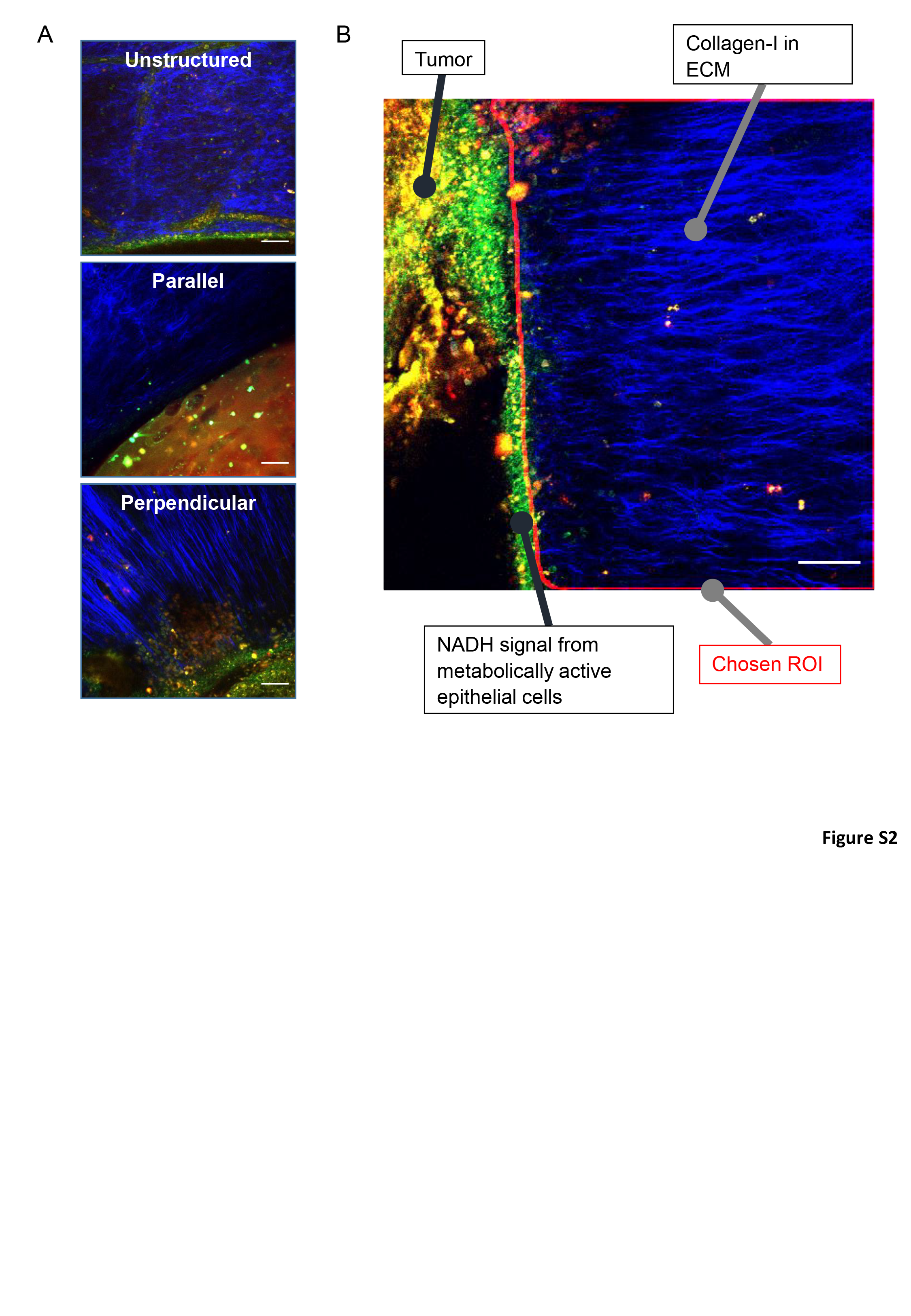

Supplement: Supplementary file 1 [file cancers-14-02364-s001.zip › cancers-1699830-supplementary/Figure S2(300dpi).tif]

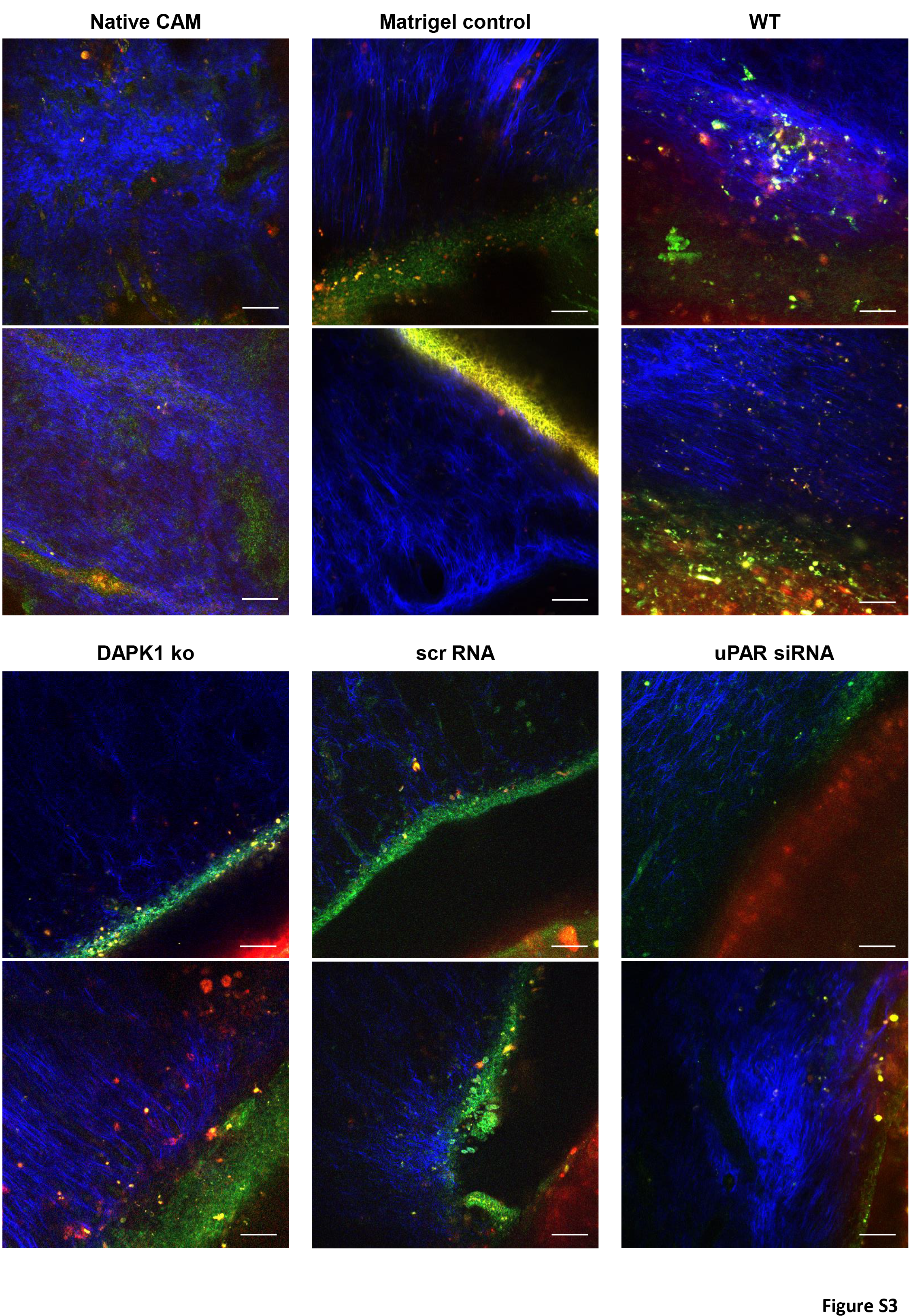

Supplement: Supplementary file 1 [file cancers-14-02364-s001.zip › cancers-1699830-supplementary/Figure S3(300dpi).tif]

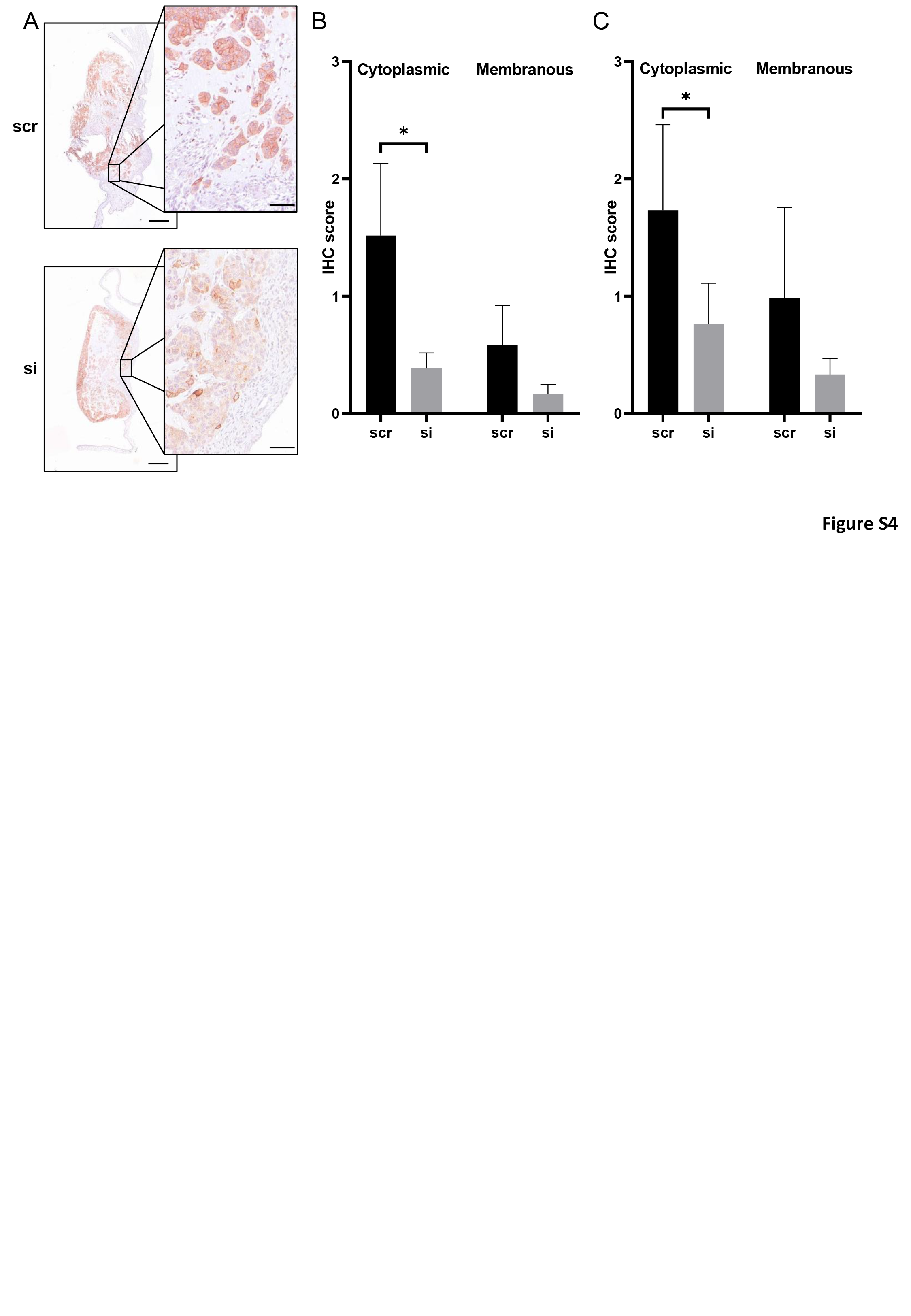

Supplement: Supplementary file 1 [file cancers-14-02364-s001.zip › cancers-1699830-supplementary/Figure S4(300dpi).tif]

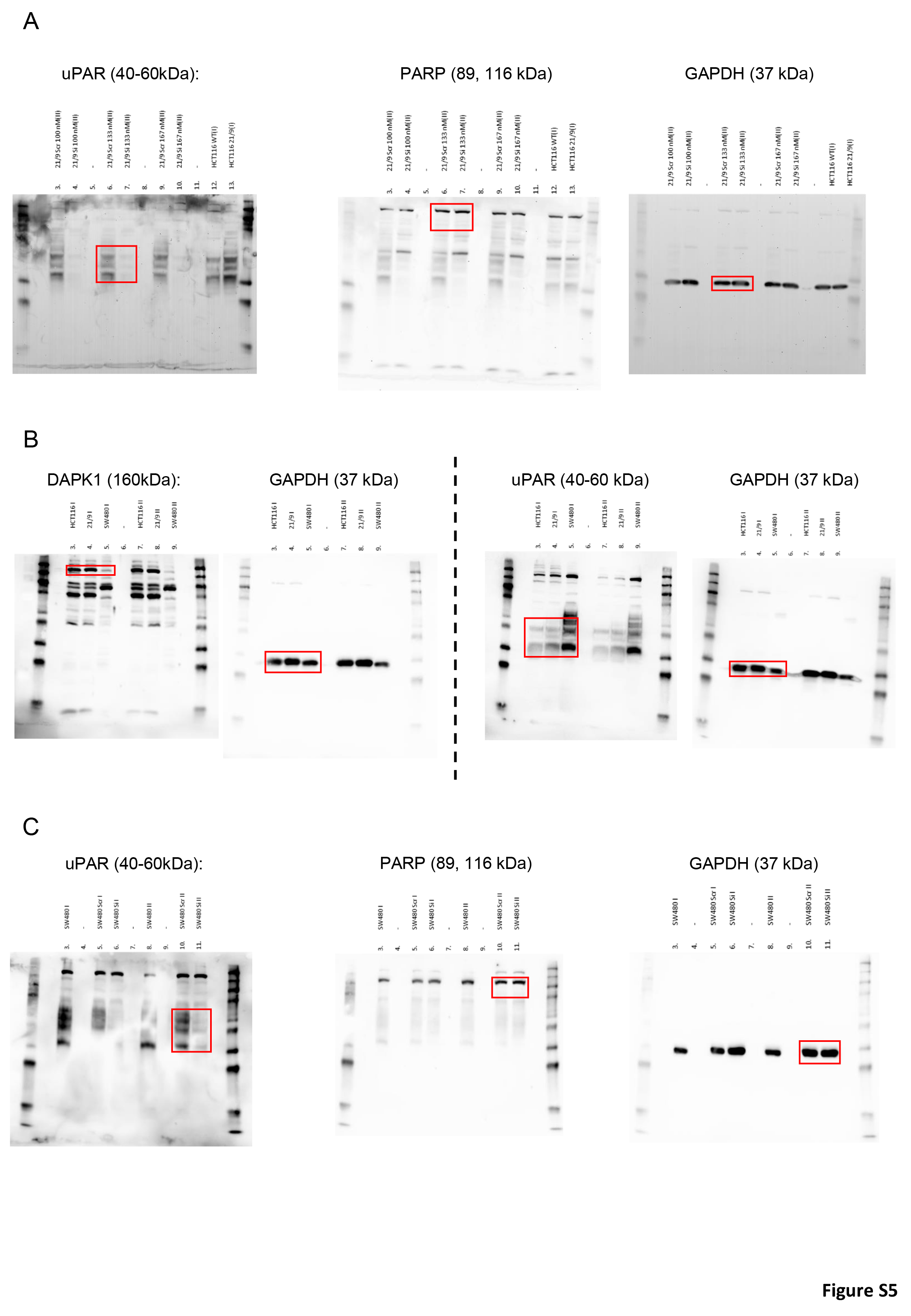

Supplement: Supplementary file 1 [file cancers-14-02364-s001.zip › cancers-1699830-supplementary/Figure S5(300dpi).tif]
